# Cross-Species Hepatic Metabolism of the Antileishmanial Chalcone NAT22 Generates Metabolites with Predicted Enhanced Affinity for the Parasite Target cTXNPx

**DOI:** 10.3390/pharmaceutics18060664

**Published:** 2026-05-27

**Authors:** Arielly R. R. Barreto, Ana Paula C. Valente, Edgar Schaeffer, Vitor M. de Almeida, Michelle F. Muzitano, Thiago Barth, Alessandra M. T. de Souza, Bárbara de A. A. Vieira, Alcides Monteiro da Silva, Osvaldo A. Santos-Filho, Patrick G. Steel, Bartira Rossi-Bergmann

**Affiliations:** 1 Instituto de Biofísica Carlos Chagas Filho, Universidade Federal do Rio de Janeiro, Rio de Janeiro 21941-902, RJ, Brazil; arielly@biof.ufrj.br; 2 Instituto de Bioquímica Médica, Universidade Federal do Rio de Janeiro, Rio de Janeiro 21941-902, RJ, Brazil; valente.anap@gmail.com; 3 Instituto de Pesquisas de Produtos Naturais Walter Mors, Universidade Federal do Rio de Janeiro, Rio de Janeiro 21941-902, RJ, Brazil; edgarippn@gmail.com (E.S.); vitoralmeida1808@ufrj.br (V.M.d.A.); osvaldo@ippn.ufrj.br (O.A.S.-F.); 4 Faculdade de Farmácia, Campus Macaé, Universidade Federal do Rio de Janeiro, Macaé 27930-560, RJ, Brazil; mfmuzitano@macae.ufrj.br (M.F.M.); barththiago@yahoo.com.br (T.B.); 5 Faculdade de Farmácia, Universidade Federal do Rio de Janeiro, Rio de Janeiro 21941-902, RJ, Brazil; amtsouza@pharma.ufrj.br (A.M.T.d.S.); barbaraabrahim@pharma.ufrj.br (B.d.A.A.V.); 6 Department of Chemistry, Durham University, Durham DH1 3LE, UK; p.g.steel@durham.ac.uk

**Keywords:** chalcone, liver microsomes, CYP, cTXNPx, leishmaniasis

## Abstract

**Background/Objectives**: Human and canine leishmaniases are neglected diseases with limited therapeutic options. The nitrochalcone NAT22, a high-affinity inhibitor of the essential parasite enzyme tryparedoxin peroxidase (cTXNPx), has emerged as a promising antileishmanial candidate. Interestingly, NAT22 demonstrated superior efficacy when administered orally rather than intralesionally, suggesting a metabolism-driven activity enhancement. Since in vivo studies with chalcones have been conducted exclusively in mice, this study aimed to determine whether mice are suitable models for oral chalcone therapies for human and canine leishmaniases and to identify metabolites with potential antileishmanial activity. **Methods**: NAT22 hepatic metabolism was investigated using in silico prediction and in vitro liver microsomal assays from rats, mice, humans, and dogs. Metabolites were identified by LC-MS/MS and NMR, and docking studies were performed against cTXNPx. **Results**: In silico analysis predicted metabolism mainly by CYP1A2, CYP2A6, CYP2C8, and CYP3A4. Seven metabolites (M1–M7) were identified by LC-MS/MS and NMR in all species except mice, whose microsomes did not generate M6. Structural analyses indicated preservation of the α,β-enone system and nitro-substituted B ring in all metabolites. Docking studies showed that metabolites M2 and M4 displayed stronger predicted binding energies than NAT22. **Conclusions**: NAT22 undergoes hepatic phase I metabolism generating two metabolites with enhanced predicted interaction with cTXNPx. The similarity between human and canine metabolic profiles supports the translational relevance of oral NAT22 therapy in leishmaniasis, while metabolites M2 and M4 emerge as candidates for validation in local treatment of cutaneous leishmaniasis.

## 1. Introduction

Chalcones are naturally occurring compounds characterized by the 1,3-diphenyl-2-propen-1-one scaffold and they exhibit a broad spectrum of biological activities, including antimalarial, antibacterial, antiviral, and anti-inflammatory effects [1,2]. Among these properties, their antileishmanial potential has been extensively investigated through in silico, in vitro, and in vivo studies, highlighting chalcones as promising scaffolds for drug development against leishmaniases [3,4,5,6,7,8,9,10,11,12].

Leishmaniases are vector-borne neglected tropical diseases (NTDs) that manifest in a wide range of clinical forms, from cutaneous leishmaniasis—the most common and disfiguring presentation—to visceral leishmaniasis, which is frequently fatal in humans and dogs [13]. Current treatments remain suboptimal. Conventional therapies are prolonged and administered parenterally, often causing significant systemic toxicity. The only oral drug available, miltefosine, is limited by its teratogenic potential [14]. For cutaneous leishmaniasis, intralesional meglumine antimoniate reduces systemic toxicity but requires repeated administration due to rapid systemic absorption associated with its high hydrophilicity, occasionally leading to systemic effects [15]. Therefore, the development of safe and effective drugs suitable for both oral and local administration remains an urgent unmet need.

**Figure 1:**
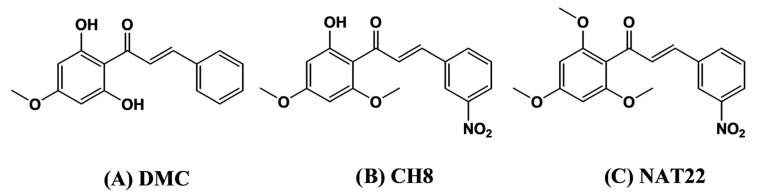
Structures of some antileishmanial chalcones.

Previously, we demonstrated the therapeutic efficacy of the natural chalcone 2′,6′-dihydroxy-4′-methoxychalcone (DMC, Figure 1A) [4] and its synthetic analogue, 3-nitro-2′-hydroxy-4′,6′-dimethoxychalcone (CH8, Figure 1B) [5], in murine cutaneous leishmaniasis using intralesional administration to minimize systemic toxicity. More recently, the analogue NAT22, which retains the antileishmanial activity of CH8 while offering a more accessible synthetic route, has become the focus of further investigation (Figure 1C).

NAT22 antileishmanial mechanism involves inhibition of tryparedoxin peroxidase (cTXNPx), an essential parasite detoxification enzyme [16]. The local therapeutic performance of chalcones has been further enhanced through the use of microparticulate controlled-release implants, enabling single-dose intralesional injection with improved efficacy compared to the free compound [10,17,18,19]. Interestingly, NAT22 demonstrated superior efficacy when administered orally rather than intralesionally, suggesting that hepatic metabolism may generate metabolites with enhanced antileishmanial activity relative to the parent compound.

Preclinical evaluation of orally administered antileishmanial agents is traditionally conducted in mice due to their ability to reproduce key features of human leishmaniasis [20]. Mice are also widely employed in drug metabolism studies because their liver microsomal cytochrome P450 (CYP) enzyme profiles exhibit substantial homology with those of humans, supporting their predictive value in metabolic investigations. In contrast, rats present a more distinct metabolic profile compared to dogs and humans [21]. Within the liver, CYP450 enzymes account for approximately 75% of phase I drug metabolism reactions [22]. However, metabolic pathways may vary considerably among species, and extrapolation of oral drug data from experimental models to target hosts must therefore be undertaken cautiously [23].

Given that in vivo metabolism studies are complex, costly, and raise ethical concerns related to the three Rs principles [24], liver microsomes provide a cost-effective and ethically preferable platform for preclinical metabolic assessment of therapeutic candidates, including alkaloids [25], lignans [26], and flavonoids [27], more specifically chalcones [28]. Previous microsomal studies of oxygenated chalcones have demonstrated that unsubstituted or mono-hydroxylated aromatic rings are susceptible to CYP-mediated hydroxylation, while methoxy substituents frequently undergo oxidative dealkylation [28,29,30]. However, the metabolic behavior of nitrochalcones remains unexplored.

In this study, we compare the hepatic metabolic profile of NAT22 using liver microsomes from four relevant mammalian species. This approach aims to evaluate the suitability of mice as a translational model for oral chalcone studies and to assess the metabolic plausibility of oral NAT22 therapy in humans and dogs. Additionally, identification of metabolites with enhanced predicted activity may contribute to the rational design of improved candidates for single-dose local treatment using appropriate delivery systems.

## 2. Materials and Methods

### 2.1. Materials

The chalcones CH8 and NAT22 were synthesized to 97% and 98% purity, respectively, as previously described [5,10]. NAT22 consisted almost exclusively (>95–98%) of the E isomer, as confirmed by ^1^H and ^13^C NMR spectroscopy and GC-MS. HPLC grade acetonitrile and methyl tert-butyl ether (MTBE) were purchased from Tedia^®^ (Fairfield, OH, USA). Potassium phosphate monobasic, hydrochloric acid, and Tris (hydroxymethyl)aminomethane were obtained from Vetec (Duque de Caxias, Brazil). Potassium hydroxide and formic acid were from Sigma-Aldrich (Saint Louis, MO, USA). Human (pooled from 50 donors), dog, rat and mouse liver microsomes (20 mg of protein/mL) were purchased from Thermo Fisher Scientific (Ward Hill, MA, USA). Glucose-6-phosphate sodium salt and nicotinamide adenine dinucleotide phosphate sodium (NADP) were from Thermo Fisher Scientific. Glucose-6-phosphate dehydrogenase was obtained from MP Biomedicals (Solon, OH, USA).

### 2.2. In Silico Prediction

ADMET PredictorTM (version 9.5, Simulations Plus, Lancaster, CA, USA) was used to predict physicochemical and ADMET properties of NAT22 and its metabolites.

### 2.3. Liver Microsomal Assay

NAT22 (12.5 µg in 125 µL acetonitrile) was pre-incubated with liver microsomal protein (5 mg in 3.3 mL of phosphate-buffered saline—PBS) from each mammalian species in a Dubnoff NT232 metabolic bath at 37 °C/10 min in duplicates. The reaction was initiated by adding 1,25 mL of a cofactor solution containing NADP^+^ (0.25 mM), glucose-6-phosphate (5 mM), and glucose-6-phosphate dehydrogenase (0.5 U/mL) prepared in Tris-HCl buffer (pH 7.4) for 90 min, making a total of 5 mL. Controls were NAT22 incubated in the absence of either cofactors or microsomal proteins. The reaction was terminated by adding 20 mL of methyl tert-butyl ether. Samples were centrifuged at 3500 rpm for 15 min at 4 °C, and 30 mL of supernatants were evaporated under nitrogen. The dried extracts were subjected to LC-MS/MS and NMR analyses, as described below.

### 2.4. LC-MS/MS Analysis

Dried microsomal extracts were reconstituted with 250 L of acetonitrile–water (3:2, *v*/*v*) for an estimated NAT22 concentration of 0.1 µg/µL, and 4 µL was injected into a reversed-phase LC-MS/MS system equipped with a Thermo Hypersil GOLD C18 column (Thermo Fisher Scientific) (50 × 2.1 mm, 1.9 µm particle size). Mobile phase A consisted of water (HPLC grade) containing 0.1% formic acid and 5 mM ammonium formate, and mobile phase B consisted of acetonitrile containing 0.1% formic acid. The analytes were eluted using a gradient starting with 15% mobile phase B that increased to 95% over 13 min and rebalanced to 15% (3 min). The flow rate was 0.35 mL/min and the column was kept at 40°C. Tandem mass spectra were acquired using positive ion electrospray with a Q Exactive Plus Orbitrap mass spectrometer (Thermo Fisher Scientific). Argon was used as the collision gas at a collision energy of 30 eV.

### 2.5. NMR Spectroscopy

The Nuclear Magnetic Resonance analyses were conducted on 600 and 800 MHz Bruker spectrometer at 25 °C at the National Center of Nuclear Magnetic Resonance Jiri Jonas (Universidade Federal do Rio de Janeiro, Rio de Janeiro, Brazil). The obtained dry microsomal extracts and standard chalcones (NAT22 and CH8) were suspended in 180 and 600 µL, respectively, of chloroform-d6. ^1^H NMR spectra were acquired using 3 mm and 5 mm TXI probes, with average of 1024 scans. Topspin (Bruker Biospin, Rheinstetten, Germany) was used for data acquisition, and the MestRENova version 6.0 was used for processing the NMR spectra.

### 2.6. Protein Modeling and Molecular Docking

Since the crystal structure of cTXNPx protein from *L. amazonensis* is not available in the Protein Data Bank (PDB), homology modeling was employed to construct a model of cTXNPx. Through the BLAST program version 2.16.0 [31], we identified a homologous protein template—specifically, the crystallographic structure of the cTXNPx protein from L. major (Uniprot: ID code: Q4VKK8) [32]. The three-dimensional model of cTXNPx was constructed using MODELLER 10.2. Molecular docking of all chalcones into the binding site was performed using AutoDock Vina 1.2.0. The structural validation of the model was carried out using the PROCHECK program. All three-dimensional images of cTXNPx, as well as chalcone–protein complexes, were generated using UCSF Chimera 1.16 program. The 2D diagrams representing the molecular interactions were generated using BIOVIA Discovery Studio Visualizer 21.1.0.20298 program.

## 3. Results

### 3.1. In Silico Prediction of Hepatic Metabolites of NAT22

**Figure 2:**
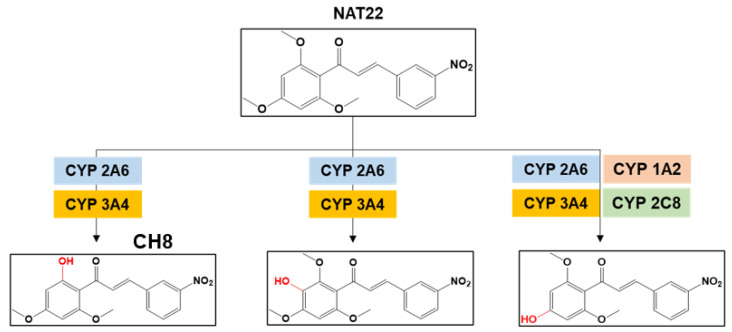
In silico prediction of NAT22 products following phase I metabolism by human cytochrome P450 (CYP) enzymes. Variations in the position of the hydroxyl groups are shown in red.

To guide and complement the in vitro metabolism studies, potential phase I metabolites of NAT22 arising from first-pass hepatic metabolism were predicted using ADMET Predictor. The cytochrome P450 (CYP) isoforms evaluated included CYP1A2, 2A6, 2B6, 2C8, 2C9, 2C19, 2D6, 2E1, and 3A4. The predictions indicated that NAT22 is primarily metabolized by CYP1A2 and CYP2A6, generating 3-nitro-3′-hydroxy-2′,4′,6′-trimethoxychalcone and 3-nitro-2′-hydroxy-4′,6′-dimethoxychalcone (CH8). Additionally, CYP1A2, 2A6, 2C8, and 3A4 were predicted to produce 3-nitro-4′-hydroxy-2′,6′-dimethoxychalcone (Figure 2).

### 3.2. Identification of NAT22 Metabolites by LC-MS/MS and NMR

**Figure 3:**
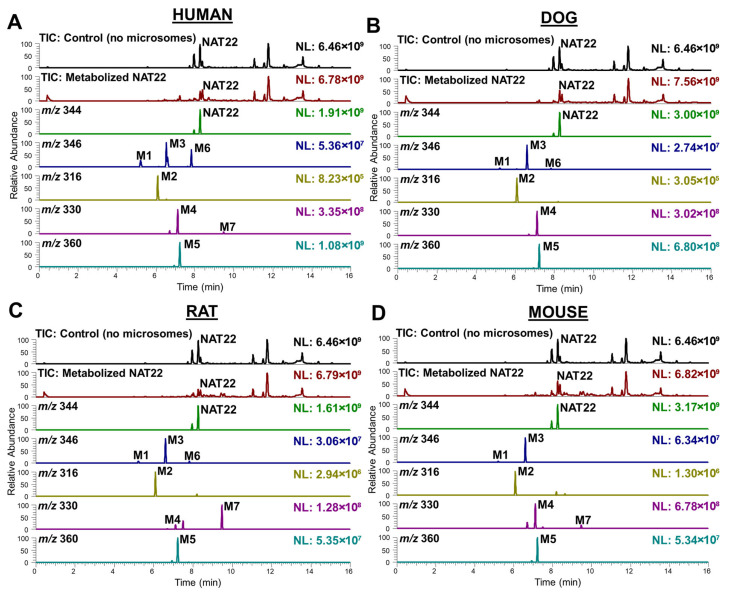
Positive ion electrospray high resolution LC-MS/MS total ion chromatogram (TIC) and computer-reconstructed mass chromatograms showing chalcone NAT22 and its phase I metabolites (M1–M7) produced after in vitro incubation with (**A**) human; (**B**) dog; (**C**) rat and (**D**) mouse liver microsomes.

NAT22 was incubated with human, dog, rat and mouse liver microsomes and the resulting extracts were analyzed by LC-MS/MS. The total ion chromatogram and computer-reconstructed selected ion chromatograms are shown in Figure 3.

Seven metabolites (M1–M7) in addition to the parent NAT22 were detected. All seven compounds were formed in human, dog and rat microsomes, whereas six (M1–M5 and M7) were detected in mouse microsomes. Exact masses and molecular formulas are summarized in Supplementary Table S1.

**Figure 4:**
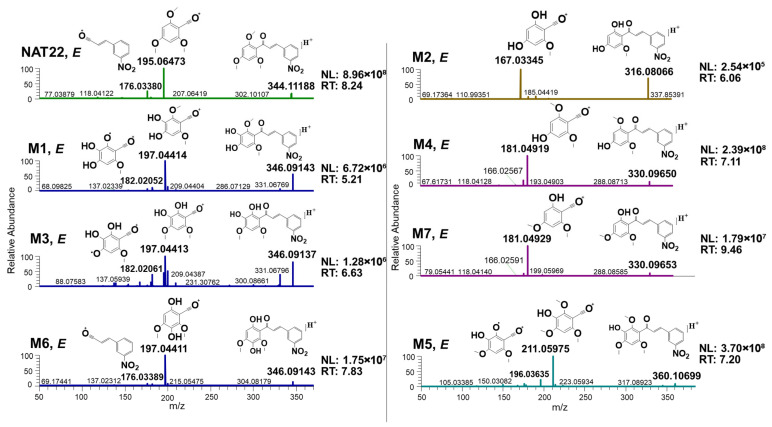
Representative positive ion ESI-MS/MS mass spectra and proposed of fragmentation of NAT22 metabolites M1–M7.

The untreated and metabolized NAT22 samples exhibited predominantly E chalcone isomers. NAT22 (*E*) presented *m*/*z* 344 ([M+H]^+^), eluting at 7.95 and 8.26 min, respectively. The CH8 standard (*m*/*z* 330, [M+H]^+^) showed Z and E isomers at 7.49 and 9.47 min. All detected metabolites predominantly corresponded to the E isomer. MS^2^ fragmentation of NAT22 generated characteristic ions at *m*/*z* 195 and 176, whereas CH8 produced ions at *m*/*z* 181 and 176. Similar acylium ion fragmentation patterns were observed for all metabolites, indicating preservation of the B ring and the *α*,*β*-enone system during metabolism (Figure 4).

**Figure 5:**
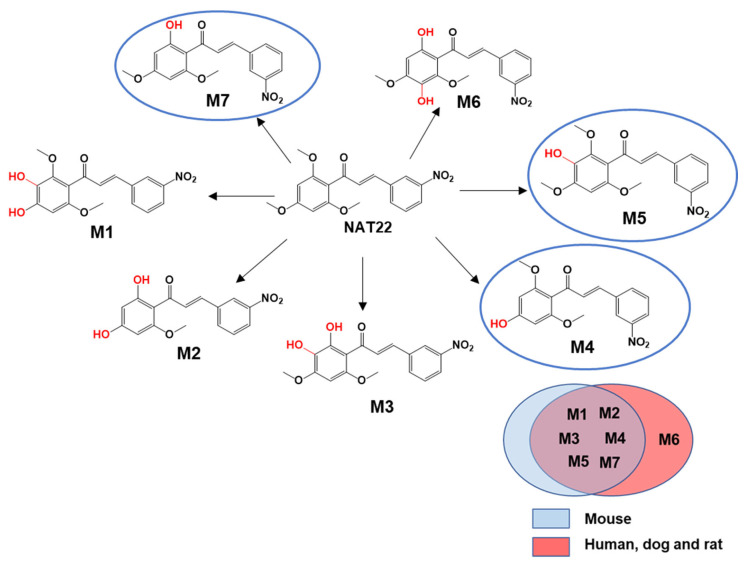
The seven metabolites that were generated following incubation of NAT22 with human, dog, rat and mouse liver microsomes. M6 was not produced by mice. Variations in the position of the hydroxyl groups are shown in red. Blue circles indicate the three metabolites that had been predicted in silico with ADMET Predictor software.

Proposed fragmentation pathways the seven metabolites (M1–M7) are represented in Figure 4, whereas their structures are in Figure 5.

Metabolites M1, M3 and M6 (*m*/*z* 346, [M+H]^+^) generated major fragment ions at *m*/*z* 197 and 182, consistent with mono-demethylation and hydroxylation on ring A. These modifications are compatible with demethylation at ortho or para methoxy groups and hydroxylation at the unsubstituted *meta* position. Metabolite M2 (*m*/*z* 316, [M+H]^+^; t_R_ = 6.08 min) showed a principal fragment at *m*/*z* 167, consistent with di-demethylation (28 Da mass loss).

Metabolites M4 and M7 (*m*/*z* 330, [M+H]^+^) yielded fragments at *m*/*z* 181 and 176, indicating mono-demethylation. M7 was confirmed as CH8 by comparison with the authentic standard. By exclusion, M4 was assigned as 3-nitro-4′-hydroxy-2′,6′-dimethoxychalcone. The shorter retention time observed for the ortho-demethylated metabolite (M7) relative to M4 is consistent with intramolecular hydrogen bonding between the ortho-hydroxyl group and the carbonyl, affecting chromatographic behavior. Based on retention time and fragmentation data, M6 (t_R_ = 7.82 min) was inferred to involve ortho demethylation combined with hydroxylation.

Metabolite M5 (*m*/*z* 360, [M+H]^+^) produced fragment ions at *m*/*z* 211 and 196, indicating hydroxylation at the meta position.

### 3.3. NMR Analysis

To confirm LC-MS/MS-based structural assignments, 1H NMR analyses were performed on NAT22 and CH8, blank samples, control incubations (without microsomes), and NAT22 incubated with human liver microsomes (37 °C, 90 min).

**Figure 6:**
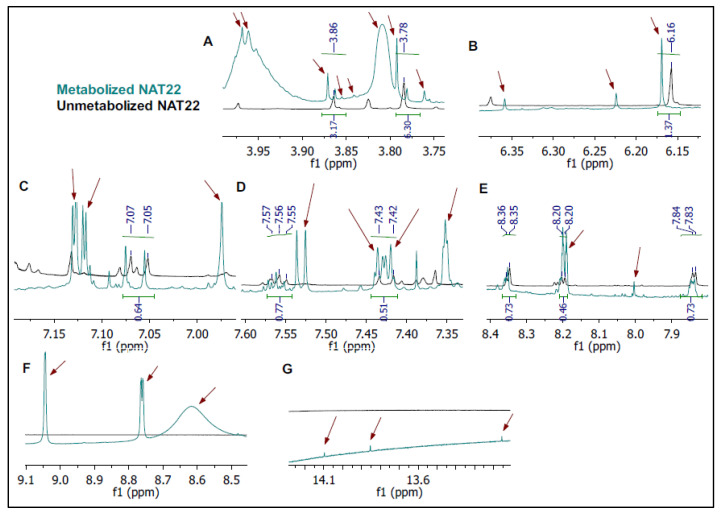
Overlay of 1H NMR spectra of NAT22 prior to (Unmetabolized) and following in vitro incubation with human microsomes (Metabolized). The blue values above and below the peaks refer, respectively, to the chemical shifts and integrals of NAT22 hydrogens in the unmetabolized control sample. The peaks indicated by arrows correspond to the hydrogen signals of the metabolites. (**A**–**G**) are the following chemical shift regions of the spectra: (**A**) Methoxy; (**B**) Aromatic (A ring); (**C**) Double bond (alfa proton); (**D**) Double bond (beta proton) and aromatic (B ring); (**E**) Aromatic (B ring); (**F**) Amine and aromatic (B ring); and (**G**) Phenolic hydroxyl.

**Figure 7:**
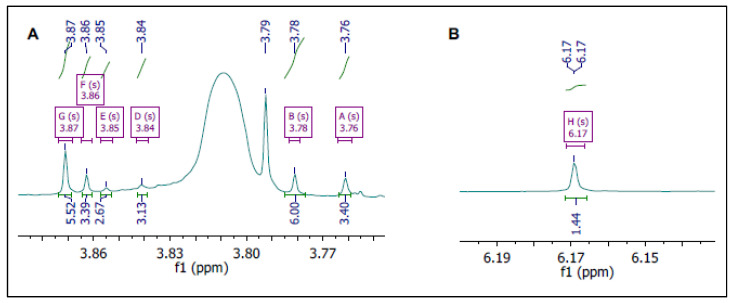
Amplification of methoxy (**A**) and aromatic (**B**) hydrogens in 1H NMR spectrum from Figure 6.

Compared with the control, the metabolized sample showed decreased signal intensity corresponding to NAT22 (Figure 6A–G) and the appearance of new resonances. In the 3.7–3.9 ppm region, four singlets (3.76, 3.84, 3.85, and 3.87 ppm) were detected, corresponding to methoxy groups in the metabolites, alongside residual NAT22 signals (3.78 and 3.86 ppm) (Figure 6A and Figure 7A). Another singlet not seen in the control could be observed at 6.17 ppm (Figure 6B and Figure 7B). Integration of signals at 3.87 ppm (s, 6H) and a corresponding 2H singlet supported assignment to M4, the only metabolite retaining symmetry in the A ring (Figure 7A). In the aromatic region (7.06–8.35 ppm) (Figure 6C–E), signal overlap with microsomal background limited detailed interpretation. However, minimal changes in B-ring proton signals were observed, consistent with preferential oxidation occurring on ring A.

Downfield signals at 13.14, 13.85, and 14.10 ppm (Figure 6G) were consistent with strongly hydrogen-bonded phenolic OH groups, supporting the presence of ortho-hydroxylated metabolites (M3, M6, and M7), in agreement with LC-MS/MS data.

### 3.4. Molecular Docking of NAT22 Metabolites

To evaluate their potential interaction with the parasite target, all metabolites were docked into a structural model of cTXNPx from *Leishmania amazonensis*. The model was based on the crystallographic structure of cTXNPx from L. major [33], with 88.4% sequence identity, and preservation of the catalytically essential Cys52 and Cys173 residues. The active site is located within a narrow cavity formed at the dimer interface, at the end of the helix that accommodates Cys-52 of one monomeric unit of the dimer and Cys-173 of the other unit [16,34].

**Figure 8:**
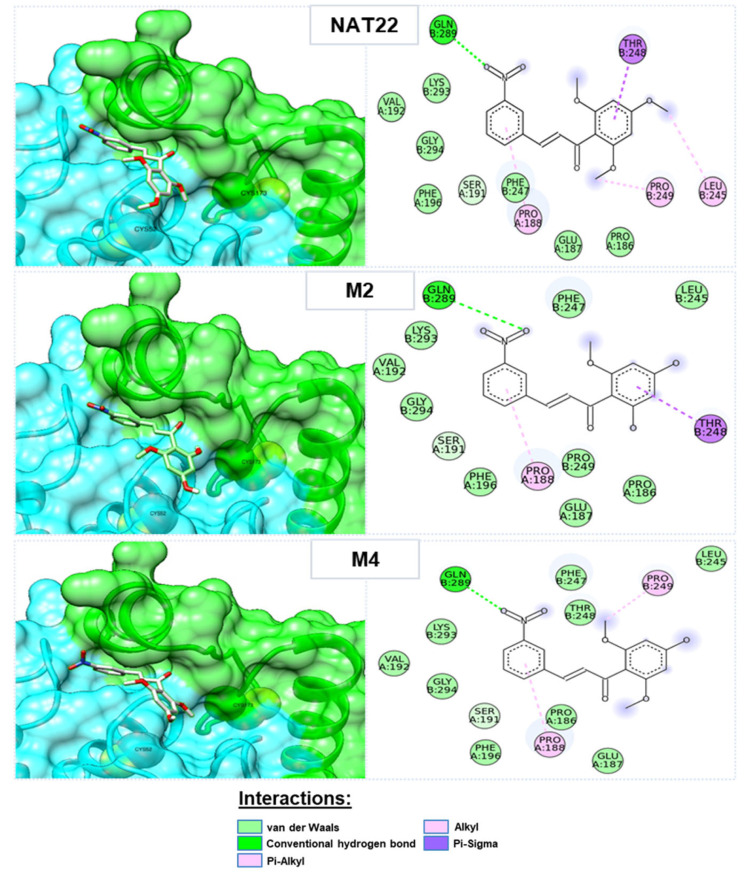
Docking of NAT22 and representative metabolites M2 and M4 between the cysteines (Cys-52 and Cys-173) of each monomeric unit of the cTXNPx enzyme. The 3D (**left**) and 2D (**right**) docking images obtained for each chalcone show the structural orientation and types of intermolecular interactions observed between the ligands and amino acids of the cTXNPx enzyme.

All metabolites were predicted to interact with the active site (Figure 8). Metabolites M2 (−6.045 kcal/mol) and M4 (−5.970 kcal/mol) showed higher predicted affinity than NAT22 (−5.964 kcal/mol) and the other metabolites (Supplementary Table S2). All chalcones formed a hydrogen bond between the nitro group and Gln289, as well as π–alkyl interactions between the B-ring aromatic system and Pro188. π-σ interactions with Thr248 were observed only for NAT22 and M1. Methoxy groups at the ortho position contributed to alkyl interactions with Pro186, Pro188, and Pro249, whereas para-methoxy groups interacted with Leu245. Notably, demethylation at ortho (M7), para (M4), or both positions (M2) altered the interaction profile toward increased van der Waals contributions, correlating with improved predicted binding energies.

## 4. Discussion

The nitrochalcone NAT22 has previously demonstrated potent antileishmanial activity in both in vitro and in vivo models [10,16,34]. However, most preclinical studies with chalcones, including those conducted in leishmaniasis, have relied on murine models. Given known interspecies differences in drug-metabolizing enzymes, extrapolation of metabolic and pharmacological data from mice to humans may not always be straightforward.

To address this limitation, we investigated the hepatic phase I metabolism of NAT22 using liver microsomes from different mammalian species. Our objectives were to characterize its metabolic profile, identify potentially active metabolites, and validate mice as suitable experimental model for therapeutic studies with chalcones. Phase I metabolism was prioritized because approximately 70–80% of drug metabolism involves oxidative, reductive, and hydrolytic reactions mediated by cytochrome P450 (CYP) enzyme family. Additionally, species-specific microsomes can serve as ethical substitutes for human volunteers and live animals, and provide reliable comparisons of metabolic profiles across species [35,36]. The chalcone NAT22 and all its metabolites are predominantly E isomers, with only a minor fraction occurring as Z isomers. As the E form is thermodynamically more stable, and given that the primary aim of this study was to compare hepatic metabolism of the chalcone across experimental and natural leishmaniasis hosts in order to predict the pharmacokinetic outcome of oral treatment, the more abundant E isomers were selected for comparative analysis. Overall, the metabolic profiles obtained in dog and rat microsomes were similar to those observed in human microsomes, since only mouse microsomes did not generate the metabolite M6. This finding suggests that murine metabolism of NAT22 may differ from the human profile to a greater extent than anticipated [21]. These interspecies differences highlight the need for caution when translating oral efficacy data obtained in mice to humans and dogs.

A good correlation was observed between in silico predictions and in vitro findings. Although only three metabolites (M7 = CH8, M5, and M4) were predicted using ADMET Predictor (Figure 2), seven phase I metabolites (M1–M7) were experimentally identified by LC-MS/MS and supported by NMR analysis. This discrepancy underscores the importance of complementing computational approaches with experimental validation. Among the seven metabolites identified, five are newly described, whereas two have been previously reported: CH8 (M7), which has documented antileishmanial activity similar to NAT22 [10], and M2, which has been associated with anticancer activity [37]. Importantly, the *α*,*β*-enone system and the nitro-substituted B ring were preserved in all metabolites, suggesting maintenance of structural features considered relevant for biological activity.

Docking studies indicated that all metabolites retained the ability to interact with the catalytic cavity of cTXNPx. Metabolites M2 and M4 showed slightly more favorable predicted binding energies compared with NAT22. Although the differences in calculated affinity were modest and require experimental confirmation, these findings suggest that phase I metabolism does not impair predicted target engagement and may subtly modulate binding interactions. The previously reported biological activity of CH8 [10], further supports the hypothesis that certain metabolites could contribute to the overall antileishmanial effect of NAT22. Nevertheless, given the pleiotropic nature of chalcones, additional molecular targets cannot be excluded.

Although further phase II metabolism in vivo is likely, the identification of phase I metabolites with preserved—or slightly enhanced—predicted affinity for cTXNPx is noteworthy.

## 5. Conclusions

This study provides the first cross-species comparative characterization of chalcone hepatic metabolism and demonstrates that phase I biotransformation of NAT22 generates metabolites with preserved or even enhanced predicted interaction with the validated parasite target cTXNPx. The identification of M2 and M4 as metabolites with stronger predicted binding affinity highlights metabolism as a potential contributor to the overall antileishmanial activity of NAT22 and supports their chemical synthesis for biological validation.

The strong concordance between human and canine metabolic profiles establishes a robust translational framework and reinforces the relevance of these species for the development of orally administered chalcone-based therapies. These findings support the continued investigation of NAT22 as an oral therapeutic candidate while also encouraging the complementary evaluation of localized delivery strategies for cutaneous leishmaniasis.

Conversely, the distinct metabolic profile observed in mice reveals meaningful interspecies differences and indicates that murine models may not reliably predict hepatic metabolism relevant to oral chalcone therapy. Importantly, this limitation may not be restricted to leishmaniasis but could extend to other disease contexts involving chalcone-based drug candidates, underscoring the need for comparative metabolism studies to strengthen translational drug development strategies.

## Data Availability

The original contributions presented in this study are included in the article/Supplementary Material. Further inquiries can be directed to the corresponding author.

## Supplementary Materials

The following supporting information can be downloaded at: https://www.mdpi.com/article/10.3390/pharmaceutics18060664/s1, Table S1: Exact masses of NAT22 and its metabolites generated by microsomes from different mammals were obtained after LC-ESI-MS/MS. Table S2: Docking energy (ordered by best interaction energy) and types of intermolecular interactions observed between the ligands and the cTXNPx enzyme.

## Abbreviations

The following abbreviations are used in this manuscript:

NAT22	3-nitro-2′-hydroxy-4′,6′-dimethoxychalcone
cTXNPx	Tryparedoxin peroxidase
CH8	3-nitro-2′-hydroxy-4′,6′-dimethoxychalcone
DMC	2′,6′-dihydroxy-4′-methoxychalcone
CYP	Cytochrome P450
LC-MS/MS	Liquid Chromatography coupled to Tandem Mass Spectrometry
NMR	Nuclear Magnetic Resonance
PBS	Phosphate-Buffered Saline
MTBE	Methyl tert-butyl ether
HPLC	High Performance Liquid Chromatography
NADP^+^	Nicotinamide Adenine Dinucleotide Phosphate
Tris-HCl	Tris(hydroxymethyl)aminomethane hydrochloride buffer
ADMET	Absorption, Distribution, Metabolism, Excretion, and Toxicity
BLAST	Basic Local Alignment Search Tool
PDB	Protein Data Bank
TXI probe	Probe type for NMR spectroscopy (1H NMR)
