# Supplementary material for: Cross-Species Hepatic Metabolism of the Antileishmanial Chalcone NAT22 Generates Metabolites with Predicted Enhanced Affinity for the Parasite Target cTXNPx
Source: Pharmaceutics. 2026 May 27;18(6):664. doi: 10.3390/pharmaceutics18060664 (PMC13306700; doi:10.3390/pharmaceutics18060664)
Supplement: Supplementary file 1 [file pharmaceutics-18-00664-s001.zip › pharmaceutics-4205652-supplementary.pdf]

Table S1: Exact masses of NAT22 and its metabolites generated by microsomes from different mammals were obtained after LC-ESI-MS/MS.

Table S2: Docking energy (ordered by best interaction energy) and types of intermolecular interactions observed between the ligands and the cTXNPx enzyme.

Supplementary Table S1: Exact masses of NAT22 and its metabolites generated by microsomes from different mammals were obtained after LC-ESI-MS/MS.

**Analyte (*E*)**	**Accurate mass (m/z)**	**Human**		**Dog**		**Rat**		**Mouse**		**Molecular Formula**
		**Exact mass (m/z)**	**Error (ppm)**	**Exact mass (m/z)**	**Error (ppm)**	**Exact mass (m/z)**	**Error (ppm)**	**Exact mass (m/z)**	**Error (ppm)**	
**NAT22**	344,11286	344,11230	1,63	344,11206	2,32	344,11191	2,76	344,11218	1,98	$C_{18}H_{17}NO_6$
**M1**	346,09213	346,09186	2,92	346,09149	1,85	346,09186	0,78	346,09171	1,21	$C_{17}H_{15}NO_7$
**M2**	316,08156	316,08112	1,39	316,08099	1,80	316,08081	2,37	316,08145	0,35	$C_{16}H_{13}NO_6$
**M3**	346,09213	346,09189	0,69	346,09143	2,02	346,09140	2,11	346,09164	1,42	$C_{17}H_{15}NO_7$
**M4**	330,09721	330,09680	1,24	330,09641	2,42	330,09637	2,54	330,09650	2,15	$C_{17}H_{15}NO_6$
**M5**	360,10778	360,10745	0,92	360,10690	2,44	360,10687	2,53	360,10709	1,92	$C_{18}H_{17}NO_7$
**M6**	346,09213	346,09180	0,95	346,09140	2,11	346,09131	2,37	-	-	$C_{17}H_{15}NO_7$
**M7**	330,09721	330,09708	0,39	330,09644	2,33	330,09659	1,88	330,09677	1,33	$C_{17}H_{15}NO_6$

Supplementary Table S2: Docking energy (ordered by best interaction energy) and types of intermolecular interactions observed between the ligands and the cTXNPx enzyme.

**Ligands**	***Docking* energy (kcal/mol)**	**H-bond**	**Pi-sigma**	**Pi-alkyl**	**Alkyl**	**Van der Waals**
**M2**	-6,045	Gln-289	-	Pro-188	-	Pro-186, Glu-187, Ser-191, Val-192, Phe-196, Phe-247, **Leu-245**, **Pro-249**, Lys-293, Gly-294
**M4**	-5,970	Gln-289	-	Pro-188	Pro-249	Pro-186, Glu-187, Ser-191, Val-192, Phe-196, Phe-247, Leu-245, **Thr-248**, Lys-293, Gly-294
**NAT22**	-5,964	Gln-289	Thr-248	Pro-188	Leu-245, Pro-249	Pro-186, Glu-187, Ser-191, Val-192, Phe-196, Phe-247, Lys-293, Gly-294
**M7**	-5,927	Gln-289	-	Pro-188	Leu-245	Pro-186, Glu-187, Ser-191, Val-192, Phe-196, Phe-247, **Thr-248**, Lys-293, **Pro-249**, Gly-294
**M3**	-5,886	Gln-289	-	Pro-188	Pro-188, Pro-249	Glu-187, Ser-191, Val-192, Phe-196, Leu-245, Phe-247, Thr-248, Lys-293, Gly-294
**M1**	-5,851	Gln-289	Thr-248	Pro-188	-	Pro-186, Glu-187, Ser-191, Val-192, Phe-196, Phe-247, Pro-249, Lys-293, Gly-294
**M6**	-5,841	Gln-289	-	Pro-188	Leu-245	Pro-186, Glu-187, Ser-191, Val-192, Phe-196, Phe-247, Thr-248, Lys-293, Pro-249, Gly-294
**M5**	-5,831	Gln-289	-	Pro-188	Pro-186, Leu-245, Pro-249	Glu-187, Ser-191, Val-197, Phe-196, Phe-247, Thr-248, Lys-293, Gly-294
